# Aggressive clitoral angiomyxoma mimicking bartholinits: A case report and review of the literature

**DOI:** 10.1016/j.ijscr.2025.112057

**Published:** 2025-10-15

**Authors:** Hanane Houmaid, Myriem Sali, Karam Harou, Bouchra Fakhir, Hamid Asmouki, Abderraouf Soummani

**Affiliations:** a Gynecology-Obstetrics Department, Cadi Ayyad University Faculty of Medicine and Pharmacy of Marrakech, Morocco; b Gynecology-Obstetrics Department, Mohammed VI Hospital, Caddi Ayyad University, Marrakech, Morocco

**Keywords:** Aggressive angiomyxoma, Vulvar mass, Surgical treatment, Pathological confirmation, Case report

## Abstract

**Introduction and importance:**

Aggressive angiomyxoma is a rare, slow-growing mesenchymal tumor typically found in the vulvo-perineal and pelvic region of women of reproductive age. Its benign but locally invasive nature and high recurrence rate make accurate diagnosis and management challenging. This case highlights a common diagnostic pitfall and underscores the importance of imaging and histopathological confirmation.

**Presentation of case:**

A 32-year-old woman presented to the emergency department with a painful vulvar mass initially diagnosed as bartholinitis. Magnetic resonance imaging (MRI) revealed a well-limited mass in the left labia majora extending to the clitoris. Initial surgical excision resulted in an R2 margin, confirmed by histopathology to be aggressive angiomyxoma. The patient underwent a mandatory surgical revision, achieving R0 excision. The postoperative course was uneventful, with a good outcome at the six-month follow-up.

**Clinical discussion:**

This case illustrates the propensity for aggressive angiomyxoma to be misdiagnosed as more common conditions like Bartholin's gland cysts. MRI is the imaging modality of choice for characterizing the lesion and planning surgery. Complete surgical excision (R0) is the cornerstone of treatment to minimize the high risk of local recurrence. Hormonal therapy with GnRH analogues can be considered as an adjuvant treatment.

**Conclusion:**

Aggressive angiomyxoma is a rare pathology that requires a high index of suspicion. Management should be undertaken in specialized centers with expertise in soft tissue tumors to ensure complete resection and manage potential recurrences. Increased awareness among clinicians is crucial for early diagnosis and appropriate treatment.

## Highlights

• Aggressive angiomyxoma: slow-growing vulvoperineal tumor often mistaken for Bartholin cyst; MRI: T2-bright myxoid mass.
• Histopathology confirms diagnosis; surgery is first-line.R0 resection is key to lowering risk of local recurrence.
• Multidisciplinary care recommended; GnRH analogues may be adjuvant therapy for recurrences or selected primary cases.
• Consider rare tumors in the differential of common vulvar swellings to prevent diagnostic delay in similar presentations.

## 1 Introduction

Aggressive angiomyxoma is a rare, slow-growing mesenchymal tumor that predominantly occurs in the vulvo-perineal and pelvic region of women of childbearing age [1,2]. Despite its benign histology, it is characterized by local invasiveness and a high propensity for recurrence if not completely excised [3]. Clinically, it is often unrecognized or misdiagnosed as a more common entity like a Bartholin's gland cyst or a lipoma [4]. Preoperative suspicion is crucial as it significantly modifies surgical planning, aiming for wide local excision to improve the patient's prognosis [3]. Recognition of its typical features on Magnetic Resonance Imaging (MRI) is therefore essential for guiding management. The gold standard treatment is complete surgical excision [5]. We report a case of aggressive clitoral angiomyxoma that was initially misdiagnosed as bartholinitis. This case report has been reported in line with the SCARE 2023 criteria [20], which provide a standardized framework for reporting surgical cases to ensure completeness and transparency. This case underscores the importance of considering rare tumors in the differential diagnosis of common vulvar swellings to avoid diagnostic delay.

## 2 Case report

### 2.1 Patient information & history

A 32-year-old woman, G2P2, with no significant past medical or surgical history, presented to the gynecologic emergency department with a large, painful vulvar swelling that had been initially misdiagnosed as bartholinitis by her primary physician. She reported no history of allergies and was not on any regular medication.

### 2.2 Clinical findings

**Fig. 1:**
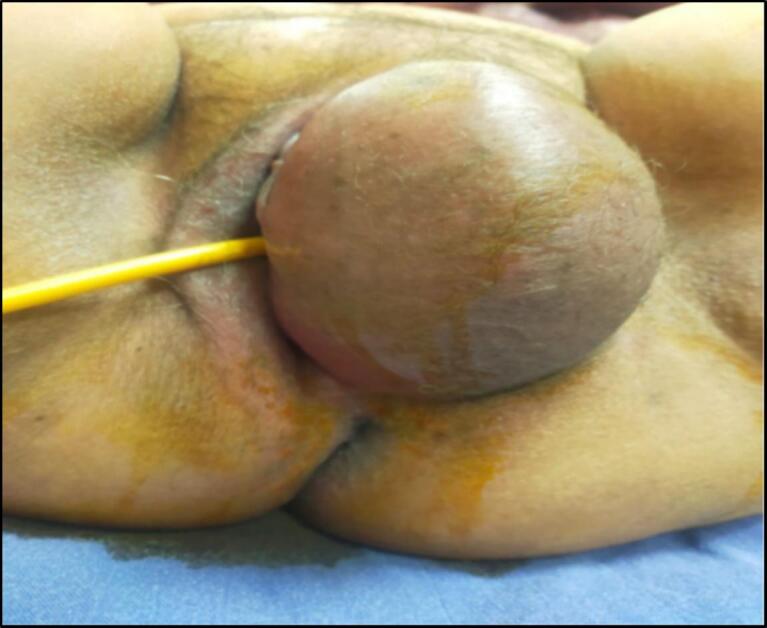
Aggressive pelvic angiomyxoma with trans-vulvar extrusion misdiagnosed as bartholinits.

On clinical examination, a large swelling was observed extending from the left labia majora to the clitoris, measuring approximately 20 cm × 18 cm × 16 cm. The mass was soft and non-fluctuant without any signs of discharge, bleeding, or skin changes (Fig. 1).

### 2.3 Diagnostic assessment & interpretation

**Fig. 2:**
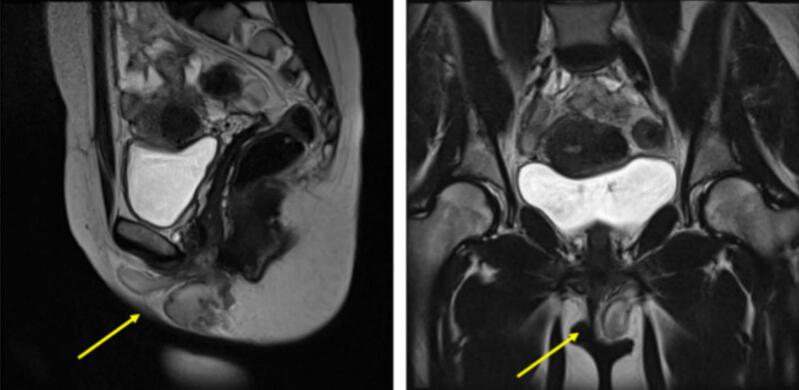
Sagittal and coronal T2 pelvic MRI: hypersignal myxoid mass of the left labia majora extending upwards into the ischio-rectal fossa, with no mass effect on adjacent organs.

A soft tissue ultrasound was initially performed and misinterpreted as a large vulvar collection or cystocele. Subsequently, a pelvic MRI was conducted for further characterization. The MRI revealed a well-circumscribed mass originating from the left labia majora and extending to the clitoris. The mass measured 7 cm on its longest sagittal axis, abutted the left pelvic floor, and extended discreetly into the ischio-rectal fossa. It demonstrated typical characteristics of aggressive angiomyxoma: T1 hyposignal, T2 hypersignal (Fig. 2), and homogeneous intense enhancement after contrast administration, with a subtly striated internal appearance.

The primary differential diagnoses included bartholinitis, Bartholin's gland cyst, and other soft tissue tumors. The imaging characteristics, particularly the high T2 signal and enhancement pattern, were highly suggestive of aggressive angiomyxoma and not typical of an infectious process like bartholinitis.

### 2.4 Therapeutic intervention & histopathological findings

**Fig. 3:**
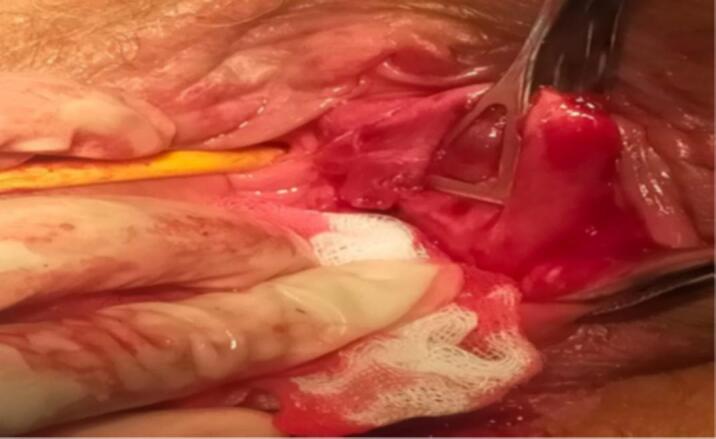
Surgical excision of the tumor.

The patient underwent initial surgical excision with a presumptive diagnosis of a Bartholin's gland cyst. The histopathological examination of the excised specimen confirmed the diagnosis of aggressive angiomyxoma. However, the excision margins were involved, classified as an R2 resection (indicating macroscopic residual tumor) (Fig. 3).

Given the high risk of recurrence, a surgical revision was performed three weeks later. During this second procedure, a wide local excision was achieved with clear microscopic margins (R0 resection).

### 2.5 Follow-up and outcomes

The postoperative recovery was straightforward. The patient was scheduled for regular clinical follow-up. A follow-up MRI at six months showed no evidence of residual disease or recurrence, and the patient remained asymptomatic.

### 2.6 Patient consent

Written informed consent was obtained from the patient for the publication of this case report and any accompanying images. A copy of the written consent is available for review by the Editor-in-Chief of this journal upon request.

## 3 Discussion

**Fig. 4:**
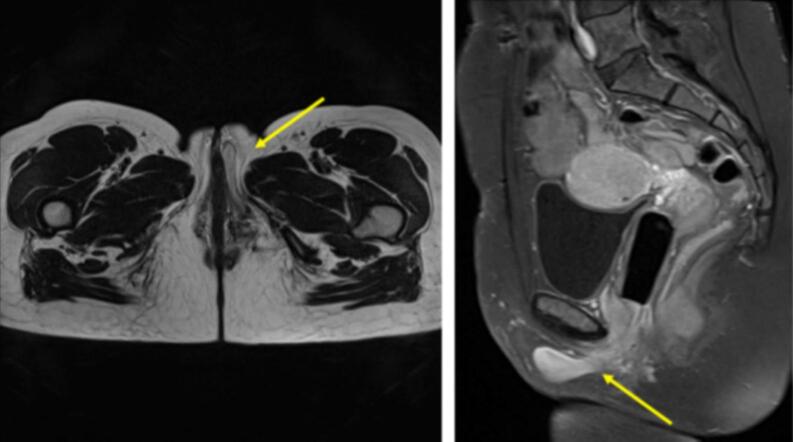
Axial T2 and sagittal T1 pelvic MRI after gadolinium injection and fat signal saturation: tumoral remnant of the labia majora adjacent to the left pelvic floor with the same characteristics as the initial tumor: T2 hyposignal and intense enhancement after gadolinium injection.

This case exemplifies the diagnostic challenges associated with aggressive angiomyxoma, a rare benign mesenchymal tumor first described in 1983 [8]. With approximately 350 cases reported, it originates from the perineum and lower pelvis, affecting women in about 90 % of cases [6,7]. These tumors are slow-growing but are termed “aggressive” due to their high local recurrence rates, which range from 9 % to 73 % depending on the completeness of the initial excision [17]. This variability can be attributed to factors such as tumor size, anatomical location (particularly deep extension across the pelvic diaphragm), and the surgical team's experience with this rare pathology [18] (Fig. 4).

The clinical presentation is often insidious. Due to its slow growth and soft consistency, patients may be asymptomatic until the tumor reaches a considerable size, as in our case. It is frequently misdiagnosed as a Bartholin's gland cyst, hernia, or lipoma [11]. Imaging is pivotal for pre-operative diagnosis. Ultrasound often shows a hypoechoic or pseudocystic mass, with Doppler potentially revealing its vascular nature. CT scan shows a hypodense mass, but MRI is the most effective modality, typically demonstrating a well-defined mass with T1 hyposignal, T2 hypersignal, and characteristic swirling or layered patterns post-contrast [6,12].

**Fig. 5:**
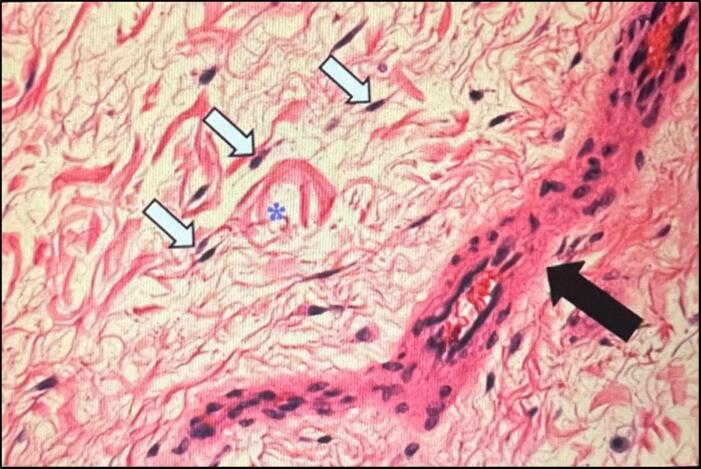
Stellate or spindle-shaped mesenchymal cells (white arrows) in a myxoid stroma (*) with collagen fibers and small vessels with thickened walls (black arrow).

Macroscopically, the tumor is unencapsulated and gelatinous. Histopathology reveals a hypocellular lesion with spindle-shaped or stellate cells dispersed in a myxoid stroma, featuring a prominent vascular component with dilated capillaries and thickened walls, without significant nuclear atypia or mitosis [15]. The expression of estrogen and progesterone receptors in some tumors suggests potential hormonal sensitivity [2] (Fig. 5).

The primary and definitive treatment is complete surgical excision with negative margins (R0 resection) [5]. Incomplete excision (R1: microscopic residual; R2: macroscopic residual) is the primary risk factor for recurrence, often necessitating re-operation [20]. Radiotherapy is ineffective due to the tumor's low mitotic activity. The hormonal sensitivity of some tumors provides a rationale for using GnRH analogues as neoadjuvant or adjuvant therapy [21]. This treatment can help reduce tumor size pre-operatively or manage recurrences, potentially avoiding mutilating surgery, especially in young women concerned about fertility preservation [16]. Management, particularly in complex scenarios like pregnancy, requires a multidisciplinary team approach. Surgery alone (e.g., vulvectomy) generally does not affect biological fertility. However, pelvic radiation therapy or chemotherapy can damage the ovaries, accelerate menopause, or reduce ovarian reserve. it indirectly affects fertility by impairing sexual function through damage to the female genital tract. [[9], [10], [11], [12], [13], [14], [15], [16], [17], [18], [19], [20]].

Postoperative monitoring is essential due to the high risk of recurrence. Long-term follow-up with clinical examination and MRI is recommended to detect recurrences early, which typically exhibit the same radiological characteristics as the primary tumor [[13], [14], [15], [16], [17], [18], [19], [20], [21]].

## 4 Conclusion

In conclusion, aggressive angiomyxoma, despite its benign histology, presents a significant clinical challenge due to its locally infiltrative behavior and high risk of local recurrence. This case highlights the critical importance of considering this entity in the differential diagnosis of any vulvovaginal or pelvic mass in women of reproductive age, especially when the clinical presentation resembles more common conditions such as bartholinitis or a Bartholin's cyst. Maintaining a high index of suspicion is essential for timely diagnosis.

Magnetic resonance imaging (MRI) is the cornerstone of preoperative planning, providing unparalleled characterization of the lesion's extent and its relationship to critical pelvic structures. However, definitive diagnosis requires histopathological confirmation through biopsy. The cornerstone of curative treatment is complete surgical excision (R0 resection). Management of this complex tumor must be conducted at a specialized center with expertise in soft tissue tumors and pelvic oncology. This is essential for several reasons: the intricate surgical techniques needed to achieve negative margins in the deep pelvic spaces; the comprehensive understanding of the tumor's insidious growth patterns and behavior necessary for adequate dissection; and the experience in managing potential complications and providing multidisciplinary care, including collaboration with plastic and reconstructive surgeons.

The broader implication for clinical practice is the critical need to increase awareness among general gynecologists, primary care physicians, and emergency room clinicians. Early recognition and timely referral to a tertiary care center before any intervention can prevent incomplete resections that may lead to future, more complex recurrences.

Finally, given the rarity of aggressive angiomyxoma, significant questions remain. Future research should focus on standardizing treatment protocols, clarifying the definitive role and long-term efficacy of adjuvant hormonal therapies (e.g., GnRH analogues) as primary or neoadjuvant treatments, and establishing robust surveillance guidelines through multi-institutional collaborative studies. Advancing our understanding of the molecular drivers of this tumor could also unlock targeted therapeutic options, ultimately improving long-term outcomes and quality of life for affected patients.

## Author contribution

Hanane Houmaid: data collection

Myriem Sali: writing the paper

Karam Harou: data analysis

Bouchra Fakhir: interpretation

Hamid Asmouki: study concept

Abderraouf Soummani: study design

## Consent

Written informed consent was obtained from the patient for publication and any accompanying images. A copy of the written consent is available for review by the Editor-in-Chief of this journal on request.

## Ethical approval

Ethical approval for this study (Ethical Committee N° EC 1302) was provided by the Ethical Committee of Cadi Ayyad University Faculty of Medicine and Pharmacy of Marrakech MOROCCO on 06 February 2025.

## Guarantor

Hanane Houmaid.

## Research registration number

1. Name of the registry: Aggressive clitoral angiomyxoma mimicking bartholinits: a case report and review of the literature
2. Unique identifying number or registration ID: researchregistry11113
3. Hyperlink to your specific registration (must be publicly accessible and will be checked): https://www.researchregistry.com/browse-theregistry#home/registrationdetails/67de1eabe7f9d002bd196df9/

## Funding

This research did not receive any specific grant from funding agencies in the public, commercial, or not-for-profit sectors.

## Conflict of interest statement

The authors declare that they have no conflicts of interest regarding the publication of this case report.
